# Lipid-Enriched Diet Advances Early Oocyte Development in the Endangered *Leptobotia elongata*: Metabolomics-Based Insights into Ovarian Metabolic Remodeling

**DOI:** 10.3390/ani16142199

**Published:** 2026-07-15

**Authors:** Yuxin Jiang, Yihui Mei, Lin Luo, Jian Gao, Min Guan, Xiaojuan Cao

**Affiliations:** 1 College of Fisheries, Engineering Research Center of Green Development for Conventional Aquatic Biological Industry in the Yangtze River Economic Belt, Ministry of Education, Huazhong Agricultural University, Wuhan 430070, China; 18744284955@163.com (Y.J.); myh@webmail.hzau.edu.cn (Y.M.); gaojian@mail.hzau.edu.cn (J.G.); 2 Hubei Key Laboratory of Three Gorges Project for Conservation of Fishes, Yichang 443100, China; luolin20210618@163.com; 3 Chinese Sturgeon Research Institute, China Three Gorges Corporation, Yichang 443100, China

**Keywords:** *Leptobotia elongata*, ovarian development, dietary lipid supplementation, long-chain polyunsaturated fatty acids, phospholipid remodeling

## Abstract

*Leptobotia elongata*, an endangered freshwater fish endemic to the upper Yangtze River, increasingly depends on artificial reproduction for conservation. However, a captive juvenile population fed a conventional diet commonly exhibits ovarian development arrested at stages I–II, representing a critical bottleneck for captive reproduction. To address this, juvenile *L. elongata* were reared for 6 months on either a basal diet (control group, CG) or a lipid-enriched diet in which soybean oil was replaced by fish oil and soybean lecithin, with vitamin E added (treatment group, TG). Histological examination revealed that TG ovaries advanced from stage I–II to stage III, with markedly larger oocytes and centralized nucleoli. LC-MS/MS-based untargeted metabolomics profiling identified 1773 metabolites, of which 566 differed significantly between groups (374 up- and 192 down-regulated in TG). KEGG enrichment revealed 15 significantly perturbed pathways, with nucleotide metabolism showing the highest enrichment (Rich factor = 0.22), accompanied by changes in fatty acid biosynthesis, biosynthesis of unsaturated fatty acids, glycerophospholipid metabolism, aminoacyl-tRNA biosynthesis, pantothenate and CoA biosynthesis, and TCA-related modules. Coordinated up-regulation of long-chain polyunsaturated fatty acids (DHA, EPA, ARA, α-linolenic acid), DHA-phosphatidylethanolamine, phosphatidylcholine and CDP-choline, together with depletion of their precursors (sn-glycerol-3-phosphate, LPI(20:4), pyrophosphate), was consistent with enhanced phospholipid remodeling and Lands-cycle activity. A multi-tier regulatory network was constructed, anchored on three pathway hubs (nucleotide metabolism, pyrimidine metabolism and fatty acid elongation) and five functional modules. This network indicated that dietary lipid supplementation was associated with coordinated changes across four interconnected metabolic axes: lipid supply and membrane remodeling, nucleotide synthesis and turnover, amino acid–protein translation, and energy–coenzyme metabolism. Collectively, these findings propose a metabolic framework and identify candidate biomarkers (e.g., DHA-PE, PC) for optimizing feed formulations of *L. elongata*.

## Simple Summary

Ensuring oocyte quality remains a paramount challenge in ex situ conservation programs, where success is critically contingent upon optimal maternal nutritional provisioning. The endemic *Leptobotia elongata* is increasingly reliant on conservation aquaculture due to wild scarcity. Yet, captive individuals suffer from impaired reproductive performance, characterized by arrested oocyte development. Because oocyte formation requires substantial amounts of specific lipid classes, we tested whether a lipid-enriched diet could promote early ovarian development. Juvenile fish received either an ordinary feed or the same feed enriched with fish oil, soybean phospholipid and vitamin E and were monitored for half a year. The enriched feed clearly advanced the eggs into a more mature growth phase. To learn why, we measured the many natural chemicals produced inside the ovaries and tracked how they changed. The optimized diet elicited a dual response in oogenesis: it markedly augmented the deposition of critical phospholipids in the oolemma and supported the biosynthetic machinery required for sustained nutrient provisioning to the developing oocyte. These results provide preliminary nutritional guidance and candidate metabolic markers that may assist the captive husbandry and conservation of this vanishing species; their effects on full maturation, spawning and offspring quality remain to be confirmed.

## 1. Introduction

The Yangtze loach (*Leptobotia elongata*) is a large benthic freshwater fish endemic to the upper and middle reaches of the Yangtze River in China, long valued for its delicate flesh and high commercial value. The Yangtze basin supports exceptional freshwater fish diversity, much of it endemic and increasingly imperilled [1]. Mirroring global declines in freshwater biodiversity [2], these species face mounting pressure from overfishing, habitat degradation and hydrological alteration, including impoundment by the Three Gorges Dam [3]. Artificial reproduction underpinned by effective broodstock management has therefore become essential for their conservation [4]. Yet captive juveniles commonly exhibit arrested ovarian development, rarely advancing beyond early oogenic stages, which is the central bottleneck addressed in the present study.

In teleosts, ovarian maturation is a process with exceptionally high lipid demand: oocytes must accumulate large quantities of fatty acids, phospholipids and lipid-soluble antioxidants to support vitellogenesis and subsequent embryonic development [5,6]. Among the commonly used dietary lipid sources, fish oil is rich in long-chain n-3 polyunsaturated fatty acids—particularly docosahexaenoic acid (DHA) and eicosapentaenoic acid (EPA)—which serve both as essential structural components of the oocyte membrane and as precursors of signalling molecules involved in steroidogenesis and ovulation [7,8]. Soybean oil, used as the lipid source of the basal (control) diet in the present study, supplies n-6 and n-3 fatty acid precursors that help balance the fatty acid composition of broodstock diets. Soybean lecithin, as the principal dietary source of phospholipids, accelerates gonadal lipid deposition and improves oocyte quality [9]. Vitamin E, the most potent lipid-soluble antioxidant, protects polyunsaturated fatty acids from peroxidative damage and stabilizes the oocyte membrane [10]. Although the individual reproductive roles of these lipid components in fish have been well established, the integrated effects of a multi-component lipid enrichment on ovarian development in *L. elongata*, and the metabolic pathways involved, have not yet been reported.

Metabolomics directly reflects the physiological state of tissues and has emerged as a powerful tool for resolving the molecular mechanisms by which nutrition modulates reproductive physiology in aquatic animals [11,12]. In the present study, the soybean-oil lipid source of the basal diet was replaced by fish oil and soybean lecithin, with vitamin E added, and a feeding trial demonstrated that this formulation promoted early ovarian development in *L. elongata*. To delineate the underlying metabolic changes, untargeted metabolomics was employed to identify differential metabolites, characterize the metabolic pathways in which they were enriched, and construct a regulatory network of lipid-induced ovarian development, thereby providing a metabolic assessment of how dietary lipid enrichment influences ovarian development in this endangered species. The findings are expected to provide metabolomics-based insights to inform the optimization of juvenile-specific feed formulations and the conservation-oriented artificial reproduction of *L. elongata*.

## 2. Materials and Methods

### 2.1. Ethics Statement

All experimental protocols in this study were approved by the Animal Experimental Ethical Inspection of Laboratory Animal Center, Huazhong Agricultural University, Wuhan, China (HZAUFI-2022–0020, 4 September 2022). All efforts were made to minimize the suffering of the fish.

### 2.2. Diet Preparation

The experimental diets were prepared as described previously [13]. Briefly, the control group (CG) was fed the basal diet, and the treatment group (TG) was fed the basal diet supplemented with fish oil, soybean lecithin and vitamin E. Specifically, the basal (control) diet contained 6% soybean oil as the supplemental lipid source, whereas in the treatment diet, the soybean oil was replaced by 6% fish oil and 6% soybean lecithin, with 0.05% vitamin E added as an antioxidant and the dextrin content adjusted accordingly to keep the two diets isonitrogenous and approximately isoenergetic. The complete ingredient formulation and proximate composition (dry matter, crude protein, crude lipid and ash) with all ingredients and chemical analyses are as reported previously [13]. All diets were manufactured into pellets (approximately 2 mm in diameter) following conventional feed processing protocols. The full ingredient list and proximate composition follow our previously reported design for *L. elongata* [11]. While detailed fatty acid profiles and α-tocopherol concentrations were not experimentally determined in the current study, the formulation dictates distinct lipid characteristics between diets: the MIX diet is expected to be enriched in n-3 PUFAs (EPA and DHA from marine fish oil) and phosphatidylcholine (from soybean phospholipids), whereas the CON diet derived its lipids predominantly from n-6 linoleic acid (soybean oil). The inclusion of 0.05% vitamin E in the MIX diet provides a substantially higher level of α-tocopherol compared to the CON diet, which relied solely on the basal tocopherols naturally present in ingredients such as fish meal and squid meal.

### 2.3. Blinding Procedure

To minimize observer bias, this study adopted a strict single-blind protocol. Group allocation information was restricted exclusively to the principal investigators (X.J.C. and M.G.) for the duration of the trial. Personnel in charge of routine husbandry tasks—namely, daily feeding, water quality monitoring, and fish health surveillance (Y.H.M., L.L., and J.G.)—remained unaware of which treatment each tank received; to support this, the two experimental diets were dispensed into visually indistinguishable containers marked solely with neutral tank codes that conveyed no compositional information. Likewise, the team members involved in ovarian sampling, paraffin sectioning, and H&E staining (X.Y.J., H.Y.M., and L.L.) carried out all outcome assessments without knowledge of group identity. Finally, data processing and statistical analyses were performed by Y.X.J. and Y.H.M. using anonymized sample identifiers, with the corresponding group labels disclosed only after the primary analytical outputs had been locked.

### 2.4. Feeding Experiment

*L. elongata* was obtained from the Wudongde Fish Reproduction Base of China Three Gorges Corporation (Kunming, Yunnan, China), where the broodstock are maintained for artificial reproduction and population restoration. After 240 juvenile fish were transported to the laboratory, they were acclimated to the recirculating aquaculture system for two weeks and fed the basal diet twice daily before the start of the feeding trial. Throughout this two-week acclimation period, the fish were held in the same recirculating aquaculture system, with water temperature, dissolved oxygen, pH and photoperiod maintained at the levels subsequently used during the feeding trial (specified below). A total of 180 juvenile *L. elongata* with uniform size and good health (6.75 ± 1.42 g, 77.78 ± 5.94 mm) were randomly assigned and stocked into an indoor RAS for a 6-month feeding trial (three replicate tanks per group, 30 fish/tank). Feed was administered by hand twice daily to apparent satiation. The following water parameters were maintained: temperature 23–25 °C, dissolved oxygen ≥ 6.0 mg/L, and pH 7.2 ± 0.3. A photoperiod of 14 h light:10 h dark (14 L:10 D) was maintained. No abnormalities in feeding activity or general health condition were observed in either group over the course of the 6-month experiment. Survival was high in both treatments, with no statistically detectable difference between them (CG: 96.67%, 87/90; TG: 97.78%, 88/90; *p* > 0.05).

### 2.5. Sample Collection

Following the feeding trial, fish were fasted for 24 h before sampling. They were then anesthetized with 100 mg/L MS-222 (buffered to pH 7.0) and euthanized for ovary dissection. Ovaries were collected only from healthy females of uniform body size; from each replicate tank, three females were randomly selected, giving nine females per group. A portion of each ovarian sample was fixed in 4% paraformaldehyde (pH 7.4) for H&E histology (*n* = 9 per group), while the sample was snap-frozen for subsequent metabolomic analysis (*n* = 5 per group). Specifically, five of the nine females per group were selected so that all three replicate tanks were represented (two females from each of two tanks and one female from the remaining tank); within this constraint, all individuals were chosen randomly.

### 2.6. Histological Observation

For histological observation, the ovarian samples fixed in 4% paraformaldehyde (pH 7.4) were dehydrated through a graded ethanol series (70%, 80%, 90%, 95% and 100%), cleared in xylene, and embedded in paraffin. The paraffin-embedded tissues were cut into 5 μm thick serial sections with a rotary microtome, and the sections were mounted on glass slides and dried. After deparaffinization in xylene and rehydration through a descending ethanol series, the sections were stained with haematoxylin for 5 min, differentiated in 1% acid alcohol, blued in running tap water, and counterstained with eosin for 2 min. The stained sections were then dehydrated through an ascending ethanol series, cleared in xylene, and mounted with neutral resin. Finally, the sections were observed and photographed under a light microscope, and the developmental stages of the oocytes were classified according to their morphological characteristics. For quantitative assessment, three non-adjacent sections (≥50 µm apart to avoid double-counting) were analysed per fish (*n* = 9 per group). The entire cross-sectional area of each section was examined, and every oocyte with a clearly visible nucleus was counted and staged (I–III) based on morphology. The proportion of each stage per fish was calculated as (number at that stage/total staged oocytes) × 100%, and these per-fish values were averaged across the group. Oocyte diameters were measured with ImageJ (v1.53) as the mean of the longest and shortest axes for 20–30 oocytes per stage per fish, all sectioned through the nucleus.

### 2.7. Metabolite Extraction and LC-MS/MS Analysis

Metabolite extraction was performed on ovarian samples (25 ± 1 mg; *n* = 5 per group from TG and CG). Each sample was randomly collected from the healthy female fish with uniform body size. Samples were homogenized with beads in 500 μL of extraction solvent (MeOH:ACN:H_2_O, 2:2:1, *v*/*v*) spiked with deuterated internal standards, using a sequence of vortexing (30 s), bead-beating (35 Hz, 4 min), and sonication in a cold water bath (5 min at 4 °C); this sequence was repeated twice. Subsequently, extracts were incubated at −40 °C for 1 h to precipitate proteins and then centrifuged (12,000 rpm (≈13,800× *g*), 15 min, 4 °C). The clarified supernatant was carefully decanted into an analysis vial. Finally, a composite quality control (QC) sample was prepared by combining equal volumes of every individual supernatant.

For polar metabolites, LC-MS/MS analyses were performed using a Vanquish UHPLC system (Thermo Fisher Scientific, Waltham, MA, USA) equipped with a Waters ACQUITY UPLC BEH Amide column (2.1 mm × 50 mm, 1.7 μm; Waters, Milford, MA, USA). The column oven temperature was maintained at 40 °C. The mobile phase consisted of 25 mmol/L ammonium acetate and 25 mmol/L ammonium hydroxide in water (pH 9.7, phase A) and acetonitrile (phase B), delivered at a constant flow rate of 0.3 mL/min. The autosampler temperature was set to 4 °C, and the injection volume was 2 μL. The total run time per sample was 10 min, with the following linear gradient elution program: 0–1 min, 95% B; 1–7 min, 95% B to 65% B; 7–8 min, 65% B to 40% B; 8–8.5 min, 40% B to 95% B; 8.5–10 min, 95% B for column re-equilibration.

The UHPLC system was coupled to an Orbitrap Exploris 120 mass spectrometer (Thermo Fisher Scientific) operating in information-dependent acquisition (IDA) mode controlled by Xcalibur software (version 4.4, Thermo Fisher Scientific). Full MS scans were acquired over an m/zrange of 70–1050, with a resolution of 60,000. The top 10 most abundant precursor ions per full scan were selected for MS/MS fragmentation, with a dynamic exclusion window of 15 s to avoid redundant sequencing of identical ions. MS/MS scans were acquired over an m/zrange of 50–1050 at a resolution of 15,000. Electrospray ionization (ESI) source parameters were optimized as follows: sheath gas flow rate, 50 Arb; auxiliary gas flow rate, 15 Arb; capillary temperature, 320 °C; spray voltage, +3.8 kV in positive ion mode and −3.4 kV in negative ion mode; stepped normalized collision energy, 20/30/40.

Metabolite identification is supported by established confidence levels. Metabolite features were annotated by matching experimental MS/MS spectra against BiotreeDB (V3.0) [14], with MS1 assignments based on accurate mass and molecular formula. To convey identification confidence transparently, each annotated metabolite was assigned a confidence level according to the Metabolomics Standards Initiative [15,16]: Level 1, identity confirmed against an authentic chemical standard analysed on the same platform using at least two orthogonal properties (e.g., retention time and MS/MS); Level 2, putatively annotated on the basis of MS/MS spectral-library similarity without a reference standard; Level 3, putatively assigned to a compound class from class-diagnostic spectra or physicochemical properties; and Level 4, features detected and quantified but not identified (MS1/formula only). Because Level 2–4 annotations are not confirmed identifications, features that were biologically implausible as endogenous ovarian constituents (e.g., synthetic drugs, agrochemicals and laboratory reagents) were regarded as tentative and were not used to support biological conclusions.

### 2.8. Principal Component Analysis

Data preprocessing and multivariate analysis were performed using SIMCA software (version 18.0.1, Sartorius Stedim Data Analytics AB, Umea, Sweden). Raw data were log-transformed and mean-centered prior to automatic modeling analysis.

### 2.9. Data Preprocessing

Raw data were preprocessed using the following criteria: feature filtering was performed based on the interquartile range (IQR) to remove outliers, and features with missing values in no more than 50% of samples. Remaining missing values were imputed by substituting them with half of the minimum value detected in the dataset. Data normalization was subsequently performed based on the total ion current (TIC) of each sample. Following preprocessing, 1773 metabolites were identified at the MS/MS level. Finally, the normalized data were subjected to Pareto scaling prior to hierarchical clustering analysis and visualization as a heatmap.

### 2.10. Identification of Differential Metabolites and Hierarchical Clustering Analysis

Differential metabolites were screened using the criteria of *p* < 0.05 (Student’s *t*-test) and VIP > 1 (OPLS-DA model). The screening results were visualized in a volcano plot. For cluster analysis, a Euclidean distance matrix was computed based on the quantitative values of the identified metabolites. Hierarchical clustering was then conducted using the complete linkage method, and the clustered data were presented as a heatmap. The statistical significance (*p* value) of the top 10 down- and up-regulated differential metabolites was calculated based on their quantitative abundance data, and the distributions were visualized using box plots. The full names corresponding to the metabolite abbreviations shown in the heatmap are listed in Table A1.

### 2.11. KEGG Pathway Enrichment and Pathway Analysis of Differential Metabolites

Pathways were considered significantly enriched if they met the criterion of *p* < 0.05 (Fisher’s exact test). The Rich Factor (ratio of differential metabolites to total metabolites in a pathway) was calculated to quantify enrichment. The enrichment results were visualized in a bubble chart, where the x-axis represents the Rich Factor, the y-axis represents the pathway name, the bubble size corresponds to the number of differential metabolites, and the color gradient indicates the statistical significance (*p* value).

Metabolic pathway analysis was performed by mapping differential metabolites to authoritative databases (KEGG, PubChem) and searching the *Danio rerio* reference pathway database, the best-annotated teleost reference available for pathway-level enrichment. Enrichment results are displayed as a treemap plot, in which the size of each rectangle reflects the topological impact factor (pathway impact value) of the corresponding pathway. It was calculated as the sum of the betweenness centrality (BC) of the matched differential metabolites divided by the sum of the betweenness centrality of all metabolites in that pathway:

$$\mathrm{Pathway}\;\mathrm{Impact}=\frac{\sum_{i=1}^{w}BC_{i}}{\sum_{j=1}^{W}BC_{j}}$$

where *w* is the number of differential metabolites matched in the pathway, *W* is the total number of metabolites contained in the pathway, *BC_i_* is the betweenness centrality score of the *i*-th matched differential metabolite, and *BC_j_* is the betweenness centrality score of the *j*-th metabolite among all metabolites in the pathway.

### 2.12. Metabolomic Network Analysis

Based on the matched annotations of differential metabolites, pathway searches and regulatory interaction network analysis were performed using the most closely related species with an annotated KEGG genome, *Misgurnus anguillicaudatus*. The analysis integrated multiple layers of the metabolic hierarchy, including metabolic pathways, functional modules, enzymes, chemical reactions, and metabolites. A multi-tier “metabolite–enzyme–reaction–module–pathway” regulatory network was constructed based on KEGG annotation (Table A2). The network comprised five categories of nodes—pathway nodes, module nodes, enzyme nodes, reaction nodes and differential-metabolite (input compound) nodes—with edges representing the hierarchical affiliations or catalytic relationships annotated in the KEGG database.

### 2.13. Statistical Analysis

All quantitative data are presented as mean ± standard deviation (Mean ± SD). Statistical analyses were performed using SPSS 26.0 (IBM, Armonk, NY, USA). The normality of data distributions was assessed using the Shapiro–Wilk test, and homogeneity of variances was verified using Levene’s test. Comparisons between two groups were conducted using Student’s *t*-test. Statistical significance was defined as *p* < 0.05, and high statistical significance as *p* < 0.01.

## 3. Results

### 3.1. Histological Observation of the Ovaries

**Figure 1:**
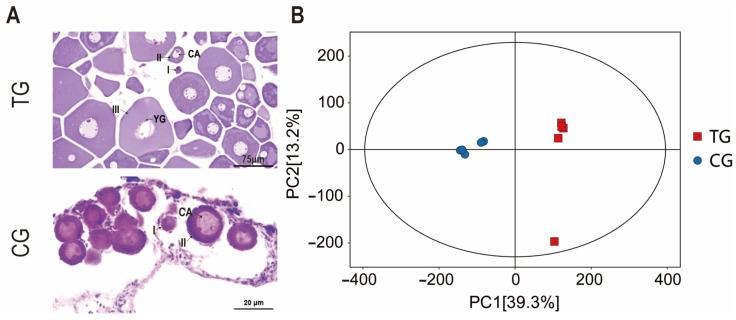
Histology of ovaries and PCA analysis of ovarian metabolomic data. (**A**) Histology of ovarian tissues from the treatment group (TG) and control group (CG). (**B**) PCA analysis of ovarian metabolomic data from TG and CG. The horizontal axis (PC1) and vertical axis (PC2) represent the scores of the first and second principal components, respectively. Each scatter point represents an individual sample. Colors and shapes denote different experimental groups. All samples fall within the 95% confidence interval (Hotelling’s T^2^ ellipse). In (**A**), oocyte developmental stages are indicated by Roman numerals (I, primary growth; II, cortical alveolus; III, early vitellogenic); ca, cortical alveoli; yg, yolk granule. The TG and CG panels are shown at different magnifications (scale bars: TG, 75 μm; CG, 20 μm).

In the control group (CG), the majority of oocytes were arrested at stages I–II (Figure 1A), with loosely arranged cells and an irregular distribution of oocytes across different developmental stages. In contrast, the TG group showed marked advancement in oogenesis, with stage III oocytes appearing exclusively in this group (they were absent in the CG); nevertheless, stage II oocytes remained the most abundant stage in both groups. These oocytes displayed distinct features, including increased cell volume and nucleoli positioned either centrally or adjacent to the nuclear envelope, reflecting a more synchronized and advanced developmental stage.

Quantitative analysis confirmed this shift (*n* = 9 females per group). In CG, the oocyte population was dominated by stage I (38.47%) and stage II (61.53%) oocytes, and stage III oocytes were absent (0%) (Table 1). In TG, the stage composition shifted markedly but was not simply reversed: stage III oocytes appeared exclusively in this group (24.90%, versus 0% in the CG) and stage I oocytes decreased to 16.37%, whereas stage II oocytes remained the most abundant stage in both groups (TG 58.77% vs. CG 61.53%) (Table 1). The diameters of stage I (CG 9.16 ± 1.58 μm vs. TG 9.06 ± 1.57 μm) and stage II (CG 17.67 ± 1.38 μm vs. TG 18.54 ± 2.13 μm) oocytes did not differ significantly between groups (*p* > 0.05). Thus, the lipid-enriched diet did not enlarge individual early-stage oocytes but instead promoted their progression into the markedly larger stage III class (77.43 ± 11.07 μm), which was unique to TG.

**Table 1:** Oocyte stage composition and diameter in the control (CG) and treatment (TG) groups.

Stage	CG Diameter (μm)	TG Diameter (μm)	CG Composition (%)	TG Composition (%)
I	9.16 ± 1.58	9.06 ± 1.57	38.47 ± 4.60	16.37 ± 2.07
II	17.67 ± 1.38	18.54 ± 2.13	61.53 ± 4.60	58.77 ± 7.46
III	n.d.	77.43 ± 11.07	n.d.	24.90 ± 5.55

Values are mean ± SD (diameter) or mean percentage of oocytes per female (composition); *n* = 9 females per group. n.d., not detected. Stage I and II diameters did not differ between groups (*p* > 0.05); stage III was present only in TG and could not be compared.

### 3.2. Global Features of the Ovarian Metabolome and Principal Component Analysis

Principal component analysis (PCA; Figure 1B) suggested a group-level separation between CG and TG samples along the first two principal components, indicating diet-associated changes in the ovarian metabolome. All five biological replicates per group are shown (*n* = 5 per group); no samples were excluded.

### 3.3. Metabolite Classification and Hierarchical Clustering Analysis

**Figure 2:**
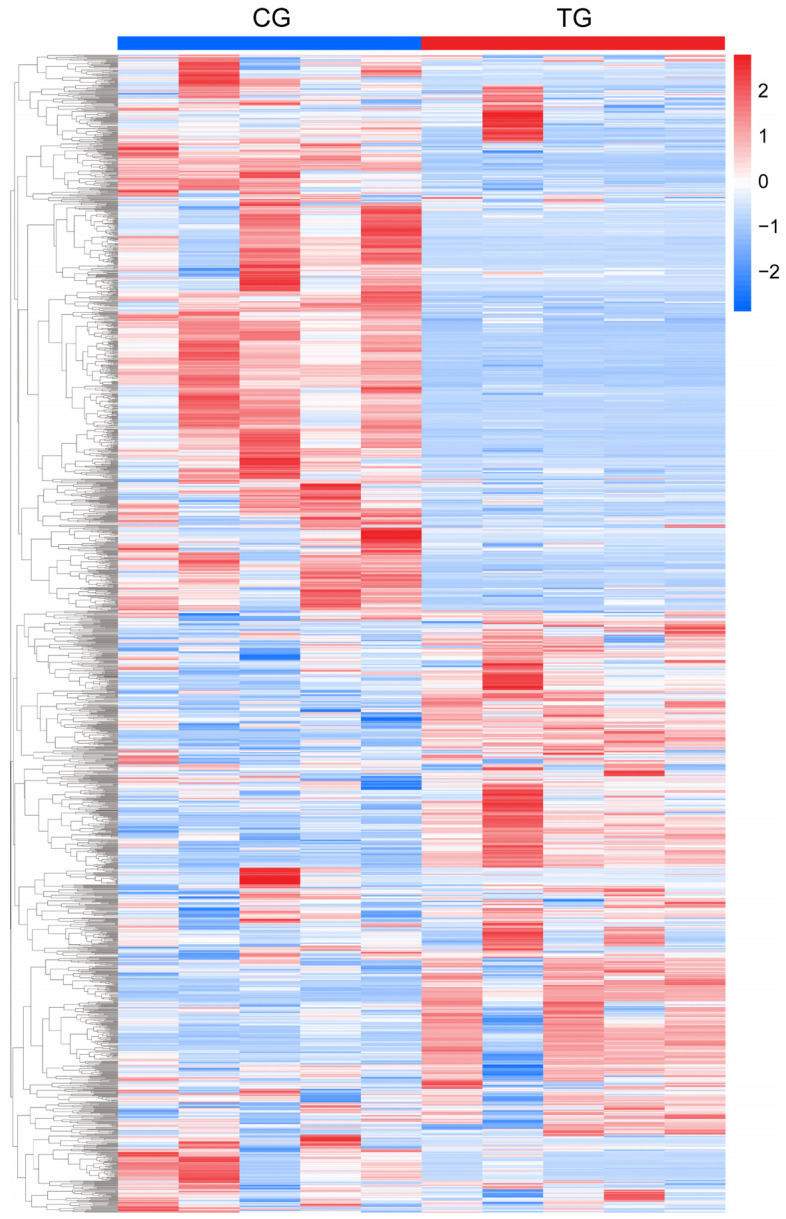
Relative abundance of all metabolites across individual samples from the treatment group (TG) and control group (CG). The x-axis represents different sample groups, while the y-axis lists all identified metabolites. The color intensity of each tile reflects the relative abundance of the corresponding metabolite, with red indicating high expression and blue indicating low expression.

The TG and CG samples were unambiguously partitioned into two distinct clusters (Figure 2), with highly similar expression patterns within each group and pronounced inter-group divergence. A substantial number of metabolites displayed concordant up- or down-regulation in the TG ovaries.

### 3.4. Identification of Differential Metabolites

**Figure 3:**
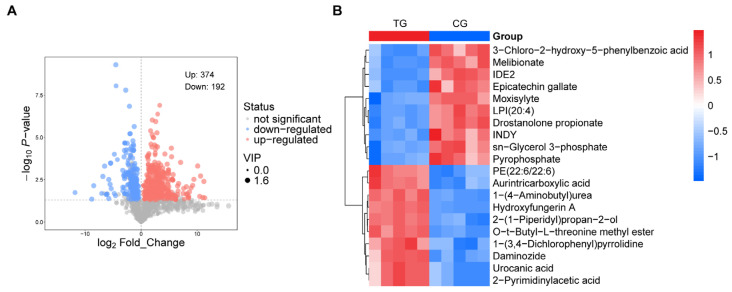
Visualization of differential metabolites: volcano plot (all metabolites) and hierarchical clustering heatmap (top 10 down/upregulated). (**A**) Volcano plot showing 374 up-regulated and 192 down-regulated differential metabolites. Each dot represents one metabolite. The size of the scatter points corresponds to the VIP value of the OPLS-DA model, with larger points indicating higher VIP values. Significantly upregulated metabolites are shown in red, significantly downregulated metabolites in blue, and non-significantly different metabolites in gray. (**B**) Relative abundance of the top 10 significantly down and upregulated differential metabolites in the treatment group (TG).

Using thresholds of VIP > 1 and *p* < 0.05, a total of 566 differential metabolites (DMs) were identified between the two groups, of which 374 were up-regulated and 192 were down-regulated in the TG (Figure 3A). The volcano plot clearly illustrates the overall distribution of these DMs: upregulated metabolites markedly outnumbered downregulated ones, and a subset displayed exceptionally high fold-changes and significance levels.

Hierarchical clustering heatmap analysis was further performed on the ten most significantly downregulated and ten most significantly up-regulated DMs (Figure 3B). These metabolites exhibited completely opposite expression patterns between the TG and CG, with pronounced between-group discrimination.

### 3.5. Quantitative Comparison of Key Differential Metabolites

It should be noted that a subset of the differentially abundant features were annotated as xenobiotic compounds (e.g., drostanolone propionate, moxisylyte, daminozide and aurintricarboxylic acid) that are not expected to occur endogenously in fish ovarian tissue. These correspond to Level 2 (putative) annotations only and most likely reflect isobaric misannotation, spectral-limitations, or trace feed/environmental signals rather than genuine biological differences; they are reported for completeness but were not interpreted further. Accordingly, the biological interpretation below focuses on metabolites with higher-confidence (Level 1) annotations and clear endogenous relevance. Quantitative comparisons were conducted on the ten most significantly downregulated and ten most significantly upregulated DMs. Given the inherent uncertainty in compound annotation from untargeted metabolomics, the following interpretation focuses on metabolites with well-defined pathway assignments and previously established relevance to oocyte development.

**Figure 4:**
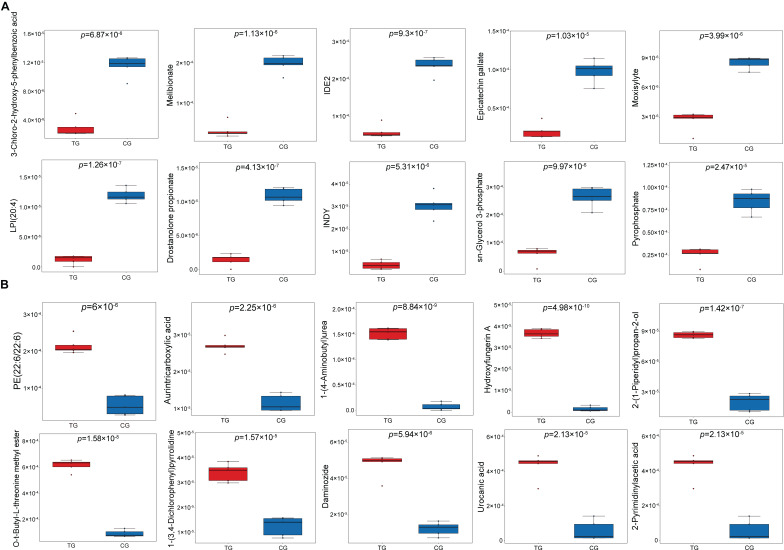
Quantitative comparison of the ten most significantly differential metabolites. (**A**) Quantitative comparison of the ten most significantly down-regulated differential metabolites in the treatment group (TG). (**B**) Quantitative comparison of the ten most significantly upregulated differential metabolites in the TG.

The metabolites significantly downregulated in the TG comprised 3-chloro-2-hydroxy-5-phenylbenzoic acid, melibionate, heptanedioic acid 1-(2-cyclopentylidenehydrazide), epicatechin gallate, moxisylyte, LPI (20:4), drostano-lone propionate, (1Z)-1-(3-ethyl-5-hydroxy-1,3-benzothiazol-2-ylidene) acetone, sn-glycerol 3-phosphate and pyrophosphate (Figure 4A). Among these, sn-glycerol 3-phosphate is the core backbone precursor for de novo glycerophospholipid biosynthesis, LPI (20:4) is an arachidonic-acid-bearing lysophospholipid intermediate of the Lands cycle, and pyrophosphate is a shared by-product of fatty acid activation and phospholipid/nucleotide biosynthesis.

The metabolites significantly upregulated in the TG comprised 1,2-di(4Z,7Z,10Z,13Z,16Z,19Z-docosahexaenoyl)-sn-glycero-3-phosphoethanolamine (DHA-PE), aurintricarboxylic acid, 1-(4-aminobutyl)urea, methyl (E)-3-{5-[(E)-4-hydroxy-3-methylbut-2-enyl]-1-methylimidazol-4-yl}prop-2-enoate, 2-(1-piperidyl)propan-2-ol, O-t-butyl-L-threonine methyl ester, 1-(3,4-dichlorophenyl)pyrrolidine, daminozide, urocanic acid and 2-pyrimidinylacetic acid (Figure 4B). Among these, DHA-PE represents the phosphatidylethanolamine (PE) form of DHA and is a key structural phospholipid of the oocyte membrane, and urocanic acid and 2-pyrimidinylacetic acid are intermediates of histidine and pyrimidine metabolism, respectively.

### 3.6. KEGG Pathway Enrichment Analysis of Ovarian Differential Metabolites

**Figure 5:**
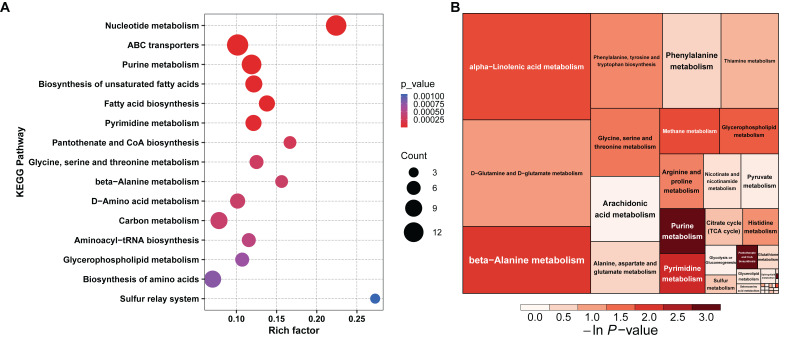
KEGG enrichment and pathway analysis of differential metabolites. (**A**) KEGG enrichment plot of differential metabolites. (**B**) Treemap of enriched metabolic pathways for differential metabolites. Each rectangle denotes a metabolic pathway; the size reflects the topological impact factor, and the color represents the enrichment significance.

Bubble plot analysis (Figure 5A) revealed that the DMs between the TG and CG ovaries were significantly enriched in 15 KEGG pathways (Fisher’s exact test, *p* < 0.05). Among these, Nucleotide metabolism showed the highest enrichment, with a Rich factor of 0.22, the smallest *p* value and the largest number of involved DMs (Count = 12), followed sequentially by ABC transporters, purine metabolism, biosynthesis of unsaturated fatty acids, fatty acid biosynthesis and pyrimidine metabolism. In addition, pantothenate and CoA biosynthesis, glycine, serine and threonine metabolism, beta-Alanine metabolism, D-Amino acid metabolism, carbon metabolism, aminoacyl-tRNA biosynthesis, glycerophospholipid metabolism, biosynthesis of amino acids, and sulfur relay system were also significantly enriched.

The hierarchical pathway treemap (Figure 5B) further corroborated these findings at higher resolution. The most pronouncedly enriched core pathways and modules included beta-Alanine metabolism, purine metabolism, pyrimidine metabolism, alpha-Linolenic acid metabolism, arachidonic acid metabolism, glycerophospholipid metabolism, citrate cycle (TCA cycle), and pantothenate and CoA biosynthesis, among others.

### 3.7. Regulatory Network Analysis of Differential Metabolites

**Figure 6:**
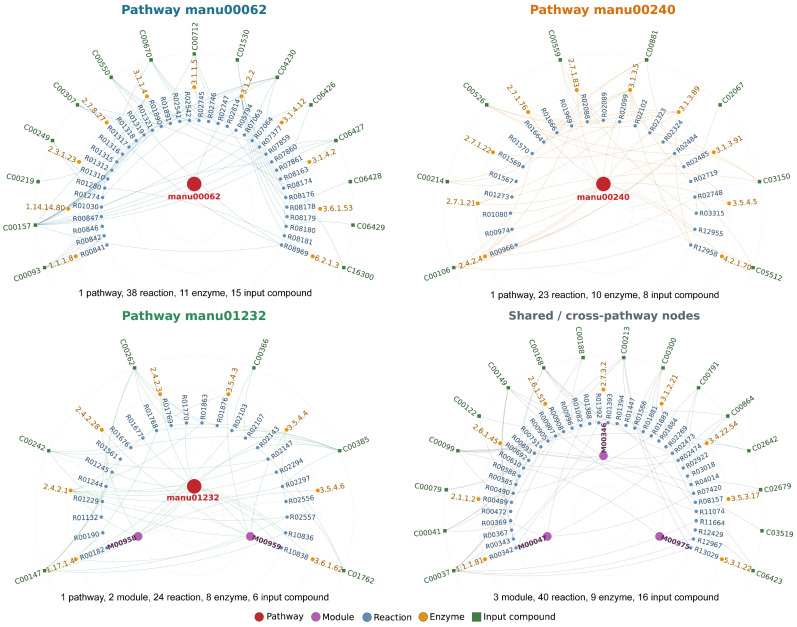
Regulatory network analysis of differential metabolites. Node colors in the regulatory network correspond to specific data types: red circles represent metabolic pathways; yellow circles represent regulatory enzymes related to the metabolites; purple circles represent molecular modules; blue circles represent chemical interaction reactions; green squares represent the differential metabolites identified in this comparison.

**Figure A1:**
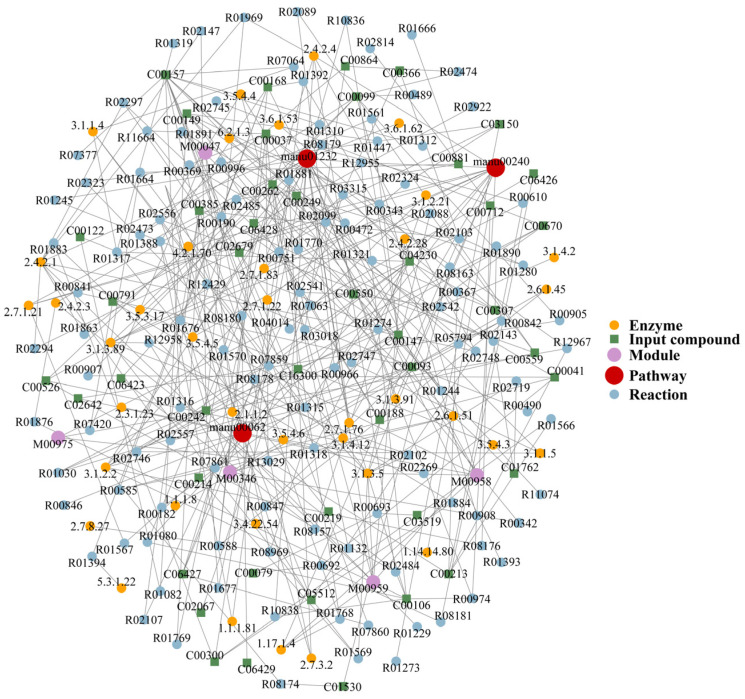
Full regulatory network analysis of differential metabolites (expanded view of Figure 6). Node colors in the regulatory network correspond to specific data types: red circles represent metabolic pathways; yellow circles represent regulatory enzymes related to the metabolites; purple circles represent molecular modules; blue circles represent chemical interaction reactions; green squares represent the differential metabolites identified in this comparison.

Network topology analysis revealed a “three-hub, multi-module” architecture centred on Nucleotide metabolism (manu01232), Pyrimidine metabolism (manu00240) and Fatty acid elongation (manu00062) (Figure 6; full network shown in Figure A1). Nucleotide metabolism exhibited the highest node connectivity, and clustered around it were purine intermediates (adenine, guanine, hypoxanthine, xanthine, uric acid) and pyrimidine intermediates (uracil, thymidine, deoxyuridine), together with enzymes such as xanthine dehydrogenase (EC 1.17.1.4), purine-nucleoside phosphorylase (EC 2.4.2.1) and thymidine kinase (EC 2.7.1.21).

Clustered around the Fatty acid elongation hub were numerous long-chain saturated, monounsaturated and polyunsaturated fatty acids, including palmitic acid, stearic acid, oleic acid, α-linolenic acid, γ-linolenic acid, stearidonic acid, arachidonic acid, EPA and DHA, which were integrated into a glycerophospholipid metabolism sub-network anchored by long-chain acyl-CoA synthetase (EC 6.2.1.3), LPCAT (EC 2.3.1.23), phospholipase A2 (EC 3.1.1.4), lysophospholipase (EC 3.1.1.5) and sphingomyelin synthase (EC 2.7.8.27) and encompassing phosphatidylcholine, CDP-choline, sn-glycero-3-phosphocholine, lysolecithin, sphingomyelin and sn-glycero-3-phosphate. Five module nodes were resolved—the Creatine pathway (M00047), Formaldehyde assimilation via the serine pathway (M00346), Adenine ribonucleotide degradation (M00958), Guanine ribonucleotide degradation (M00959) and Betaine degradation (M00975)—of which M00958 and M00959 clustered around the Nucleotide metabolism hub together with purine catabolic intermediates and their enzymes; M00346 linked nucleotide metabolism to glycine, serine and threonine metabolism; and M00047 connected creatine, creatinine, sarcosine, creatine kinase (EC 2.7.3.2) and guanidinoacetate N-methyltransferase (EC 2.1.1.2). The three hubs were interconnected through shared enzymatic nodes such as 5′-nucleotidase (EC 3.1.3.5) and phospholipase A2 (EC 3.1.1.4), bridging nucleotide and glycerophospholipid metabolism.

## 4. Discussion

Building on the developmental-arrest bottleneck in captive *L. elongata*, the present study used a lipid-enriched diet to drive the stage I–II to III transition and applied untargeted metabolomics to resolve the underlying metabolic remodeling, offering an exploratory metabolomics-based view of how dietary lipids may influence early fish ovarian development.

### 4.1. Dietary Lipids Promote Ovarian Lipid Remodeling and Oocyte Membrane Construction

In teleosts, long-chain polyunsaturated fatty acids (LC-PUFAs) are central regulators of broodstock reproduction: DHA and EPA are essential for egg and larval development, while arachidonic acid (ARA) is the precursor of reproductive eicosanoids [17]. Dietary PUFA or fish-oil supplementation accelerates maturation and improves reproductive performance across diverse taxa [18,19,20,21], and the pronounced up-regulation of multiple LC-PUFAs (DHA, EPA, ARA, α-linolenic acid) and phospholipid metabolites in TG ovaries accords with this work. A hallmark of our data is the coupling of down-regulated precursors and by-products (sn-glycerol-3-phosphate, LPI(20:4), pyrophosphate) with accumulated end-products (DHA-PE, phosphatidylcholine, CDP-choline), a pattern suggestive of enhanced phospholipid synthesis and rapid precursor turnover. This is mechanistically consistent with zebrafish tracer work, in which ^14^C-DHA is selectively shuttled to the stage-III ovary alongside up-regulated *fatp4* [22], and with the observation that *vtg1* knockout depletes ovarian DHA-PC and arrests follicle maturation, a defect partially rescued by PC and DHA co-supplementation [23]. Fish also selectively retain ovarian LC-PUFAs even under lipid-restricted diets [24,25]; the marked DHA-PE accumulation in TG ovaries thus indicates efficient incorporation of dietary DHA into membrane phospholipids.

The concurrent enrichment of phospholipase A2, lysophospholipase, LPCAT and long-chain acyl-CoA synthetase indicates coordinated activity of the Kennedy pathway and the Lands cycle [26], implying simultaneous expansion of the membrane phospholipid pool and selective enrichment of diet-derived LC-PUFAs at the sn-2 position to optimize membrane fluidity and signal-transduction capacity. The accompanying enrichment of ARA, the precursor of prostaglandins, is of particular reproductive significance: in zebrafish, ovulation is triggered through lipoxygenase-dependent eicosanoid biosynthesis [27] and ovarian ARA with its 5-LOX metabolites rises with oocyte maturation [28], while ARA supplementation enhances spawning and larval quality in other species [29]. We therefore hypothesize that the ARA enrichment seen here provides, via eicosanoid signalling, a material basis for subsequent maturation, a hypothesis that awaits direct measurement of ovarian eicosanoids and functional validation.

### 4.2. Changes in Nucleotide Metabolism Consistent with the Demand for Oocyte Genome Amplification

Nucleotide metabolism was both the most significantly enriched KEGG pathway and the most highly connected network hub, a biologically coherent result, since the stage I–II to stage III transition marks the onset of large-scale transcription, during which oocytes stockpile maternal mRNA and rRNA for vitellogenesis and early embryogenesis [5,30]. Accordingly, differential metabolites spanned purine (adenine, guanine, hypoxanthine, xanthine, uric acid) and pyrimidine (uracil, thymidine, deoxyuridine) intermediates, while enrichment of xanthine dehydrogenase, purine-nucleoside phosphorylase and adenosine deaminase indicates concurrently enhanced catabolism. This parallel synthesis–degradation reflects high-flux nucleotide turnover that supplies substrates while maintaining pool homeostasis [31], echoing the purine signalling reported in progestin-treated zebrafish ovaries [28].

Network analysis further identified module M00346 (formaldehyde assimilation via the serine pathway) as a nexus linking nucleotide metabolism to glycine, serine and threonine metabolism, underscoring the role of one-carbon provision in de novo nucleotide synthesis: serine donates one-carbon units to tetrahydrofolate for purine-ring assembly and dUMP-to-dTMP conversion [32], consistent with globally elevated nucleotide biosynthetic flux in growing oocytes.

### 4.3. Amino Acid Metabolism and the Protein Translation Machinery

KEGG enrichment and network analyses consistently revealed activation of glycine, serine and threonine metabolism, β-alanine metabolism, D-amino acid metabolism, and aminoacyl-tRNA biosynthesis in TG ovaries, aligning with the demands of stage III oocytes, which must synthesize vitellogenin receptors, cortical alveolus proteins and diverse regulatory factors in preparation for receptor-mediated vitellogenin uptake [5,33]. The enrichment of aminoacyl-tRNA biosynthesis is especially notable, as this pathway sets the rate of amino acid activation and tRNA charging that fuels ribosomal protein synthesis; comparable activation has been reported during ovarian development in the Chinese mitten crab [34]. Beyond serving as translational substrates, glycine and serine also feed one-carbon metabolism for nucleotide synthesis (Section 4.2) and drive glutathione biosynthesis for antioxidant defence [35], positioning this pathway as a hub linking nucleotide synthesis, protein translation and redox homeostasis.

This inference is directly corroborated by our companion transcriptomic analysis of the same ovaries [13]: upregulated genes were enriched in nucleolus, nuclear-pore and RNA-binding categories, and ribosome-biogenesis factors (*utp11*, *surf6*, *nop16* and *gar1*) were significantly upregulated (qRT-PCR-validated), providing convergent metabolome–transcriptome evidence for enhanced translational capacity in stage-III oocytes.

### 4.4. Energy Metabolism and Coenzyme Provision

Progression from stages I–II to stage III is energetically costly, demanding sustained ATP to drive lipid synthesis, nucleic acid and protein biosynthesis, and transport; vitellogenesis is among the most energy-demanding phases of teleost oocyte development [5]. Concomitant alterations in TCA-cycle intermediates (fumarate, malate) and enrichment of glycolysis/gluconeogenesis signify elevated central carbon flux in TG ovaries. More importantly, the accumulation of pantothenate and nicotinamide riboside signals a boost in coenzyme biosynthesis: CoA availability is rate-limiting for converting fatty acids to acyl-CoA and feeding the TCA cycle [36], while NAD^+^/NADH is the principal electron carrier of oxidative phosphorylation, together establishing a self-reinforcing cycle in which elevated CoA drives fatty acid activation to sustain phospholipid synthesis and ATP production.

Activation of the creatine–phosphocreatine shuttle (M00047) is a further notable finding: creatine kinase reversibly transfers phosphate between ATP and creatine, acting as both energy buffer and transporter linking mitochondrial ATP production to cytosolic utilization [37]. As stage III oocytes expand rapidly, efficient ATP delivery to the peripheral cytoplasm may become limiting for vitellogenesis and growth.

### 4.5. A “Three-Hub, Multi-Module” Network Suggests Coordinated Remodeling of Ovarian Metabolism

The multi-tier network revealed a highly interconnected architecture centred on three hubs—nucleotide metabolism, pyrimidine metabolism and fatty acid elongation—linked through shared nodes (5′-nucleotidase, phospholipase A2) and functional modules (creatine–phosphocreatine shuttle, one-carbon metabolism). This topology indicates that dietary lipid supplementation does not act on lipid metabolism alone but is associated through shared nodes with coordinated changes in nucleotide biosynthesis, amino acid translation and energy–coenzyme metabolism, suggesting a system-wide remodeling of the ovarian metabolic network. Such coordination is consistent with the biosynthetic burden of vitellogenesis, during which oocytes must simultaneously expand the plasma membrane, stockpile maternal RNA, synthesize vitellogenin and cortical alveolus proteins, and generate ATP [5,33]; consistent with this interdependence, *vtg1* knockout disrupts ovarian DHA-PC transport and reduces fecundity in zebrafish [23]. Combined fish oil, soybean lecithin and vitamin E supplementation appears to relieve these bottlenecks across multiple tiers, providing the material and energetic foundation for the orderly progression of oocytes to stage III. As this network was inferred from KEGG annotations rather than direct measurements, its enzyme and reaction nodes are not experimentally validated. It should be interpreted as a hypothesis-generating framework, not as evidence of enzymatic flux.

### 4.6. Potential Protective Role of Vitamin E in Ovarian Metabolic Remodeling

Although vitamin E was not itself detected among the differential metabolites, it likely contributed to the observed lipid changes. However, no oxidative-stress or lipid-peroxidation markers were measured in this study, so the antioxidant role of vitamin E inferred here remains indirect and requires targeted confirmation. As a lipid-soluble, chain-breaking antioxidant that intercalates into phospholipid bilayers and quenches lipid peroxyl radicals, vitamin E safeguards LC-PUFA-rich membranes [10]—precisely the DHA- and EPA-rich stage-III oocyte membranes that accumulate in TG ovaries and are especially vulnerable to peroxidation. Consistent with an antioxidant–PUFA synergy, supplementation in *Catla catla* shows that PUFAs combined with vitamins maximize fertilization, whereas vitamins alone cause egg-maturation failure [18]. Within the compound supplement, vitamin E is the most plausible source of this protection, although its specific contribution cannot be isolated (Section 4.7).

Independent transcriptomic evidence from the same ovaries reinforces this interpretation [13]: DNA-damage-repair and stress-response genes (*rad51*, *rad21*, *fancf* and *cdc45*) were coordinately downregulated (qRT-PCR-validated), consistent with relief of oxidative stress once the diet supplies vitamin E as a chain-breaking antioxidant alongside membrane phospholipids.

### 4.7. Limitations

A key limitation concerns the combined-treatment design: fish oil, soybean lecithin and vitamin E were administered together, so the observed responses reflect the net effect of the compound supplement rather than the contribution of any single ingredient. In addition, the dietary fatty-acid profile, gross energy and α-tocopherol content were not determined; the diets follow the formulation reported in ref. [13]. Factorial feeding trials with direct compositional analyses are needed to dissect individual contributions and refine feed targets. Furthermore, while five biological replicates per group is common in untargeted metabolomics and produced clear group separation, it limits statistical power given the large number of features detected, and cannot fully exclude false positives. Validation of candidate biomarkers (e.g., DHA-PE and PC) in larger and independent cohorts is therefore necessary before they can be applied as robust biomarkers.

## 5. Conclusions

In summary, supplementing the basal diet of juvenile *L. elongata* with fish oil, soybean lecithin and vitamin E advanced early ovarian development, carrying oocytes from the arrested stages I–II to the cortical alveolus stage (stage III) rather than to full maturation. Untargeted metabolomics showed that this advancement is underpinned by a coordinated, system-level remodeling of the ovarian metabolome, organized around a “three-hub, multi-module” architecture spanning lipid and phospholipid remodeling, nucleotide synthesis and turnover, amino acid–protein translation, and energy–coenzyme metabolism. These findings provide the first metabolomics-based characterization of lipid-associated metabolic remodeling during early ovarian development in this species and identify DHA-PE and PC as candidate biomarkers for monitoring ovarian status in captive broodstock. As advancement was documented only to stage III, and because the three components were supplied together, longer-term and factorial feeding trials will be needed to extend dietary recommendations through later maturation and to resolve each component’s contribution.

## Data Availability

The original contributions presented in this study are included in the article. Further inquiries can be directed to the corresponding authors.

## Appendix A

**Table A1:** Abbreviations of the metabolites shown in the heatmap.

Abbreviation	Full Name
IDE2	Heptanedioic acid, 1-(2-cyclopentylidenehydrazide)
LPI(20:4)	Lysophosphatidylinositol (20:4)
INDY	(1Z)-1-(3-Ethyl-5-hydroxy-1,3-benzothiazol-2-ylidene)acetone
PE(22:6/22:6)	1,2-Di(4Z,7Z,10Z,13Z,16Z,19Z-docosahexaenoyl)-sn-glycero-3-phosphoethanolamine
Hydroxyfungerin A	Methyl (E)-3-{5-[(E)-4-hydroxy-3-methylbut-2-enyl]-1-methylimidazol-4-yl}prop-2-enoate

**Table A2:** Node information of the metabolic network.

KEGG Id	Entry Type	KEGG Name	*p* Score
manu00062	pathway	Fatty acid elongation	5.12929 × 10^−5^
manu00240	pathway	Pyrimidine metabolism	8.28377 × 10^−5^
manu01232	pathway	Nucleotide metabolism	0.000001
M00047	module	Creatine pathway	0.000001
M00346	module	Formaldehyde assimilation, serine pathway	6.27648 × 10^−5^
M00958	module	Adenine ribonucleotide degradation, AMP => Urate	0.000001
M00959	module	Guanine ribonucleotide degradation, GMP => Urate	0.000001
M00975	module	Betaine degradation, bacteria, betaine => pyruvate	0.000264645
1.1.1.8	enzyme	glycerol-3-phosphate dehydrogenase (NAD+)	9.84246 × 10^−5^
1.1.1.81	enzyme	hydroxypyruvate reductase	4.40782 × 10^−6^
1.14.14.80	enzyme	long-chain fatty acid omega-monooxygenase	5.80794 × 10^−5^
1.17.1.4	enzyme	xanthine dehydrogenase	0.000001
2.1.1.2	enzyme	guanidinoacetate N-methyltransferase	0.000001
2.3.1.23	enzyme	1-acylglycerophosphocholine O-acyltransferase	0.000264296
2.4.2.1	enzyme	purine-nucleoside phosphorylase	0.000001
2.4.2.28	enzyme	S-methyl-5′-thioadenosine phosphorylase	8.98582 × 10^−6^
2.4.2.3	enzyme	uridine phosphorylase	0.000001
2.4.2.4	enzyme	thymidine phosphorylase	0.000001
2.6.1.45	enzyme	serine---glyoxylate transaminase	0.000001
2.6.1.51	enzyme	serine---pyruvate transaminase	0.000001
2.7.1.21	enzyme	thymidine kinase	0.000001
2.7.1.22	enzyme	ribosylnicotinamide kinase	0.000001
2.7.1.76	enzyme	2′-deoxyadenosine kinase	0.000001
2.7.1.83	enzyme	pseudouridine kinase	0.000001
2.7.3.2	enzyme	creatine kinase	0.000001
2.7.8.27	enzyme	sphingomyelin synthase	0.000001
3.1.1.4	enzyme	phospholipase A2	0.000001
3.1.1.5	enzyme	lysophospholipase	0.000001
3.1.2.2	enzyme	palmitoyl-CoA hydrolase	0.000001
3.1.2.21	enzyme	dodecanoyl-[acyl-carrier-protein] hydrolase	0.000001
3.1.3.5	enzyme	5′-nucleotidase	0.000001
3.1.3.89	enzyme	5′-deoxynucleotidase	0.000001
3.1.3.91	enzyme	7-methylguanosine nucleotidase	4.85225 × 10^−6^
3.1.4.12	enzyme	sphingomyelin phosphodiesterase	0.000001
3.1.4.2	enzyme	glycerophosphocholine phosphodiesterase	0.000001
3.4.22.54	enzyme	calpain-3	1.25874 × 10^−5^
3.5.3.17	enzyme	guanidinopropionase	0.000001
3.5.4.3	enzyme	guanine deaminase	0.000001
3.5.4.4	enzyme	adenosine deaminase	0.000001
3.5.4.5	enzyme	cytidine deaminase	4.69087 × 10^−6^
3.5.4.6	enzyme	AMP deaminase	0.000001
3.6.1.53	enzyme	Mn2+-dependent ADP-ribose/CDP-alcohol diphosphatase	0.000001
3.6.1.62	enzyme	5′-(N7-methylguanosine 5′-triphospho)-[mRNA] hydrolase	5.04178 × 10^−5^
4.2.1.70	enzyme	pseudouridylate synthase	0.000223198
5.3.1.22	enzyme	hydroxypyruvate isomerase	1.61876 × 10^−5^
6.2.1.3	enzyme	long-chain-fatty-acid---CoA ligase	0.000245141
R00182	reaction	AMP phosphoribohydrolase	0.000001
R00190	reaction	AMP:diphosphate phospho-D-ribosyltransferase	9.60446 × 10^−5^
R00342	reaction	(S)-malate:NAD+ oxidoreductase	0.000245808
R00343	reaction	(S)-malate:NADP+ oxidoreductase	0.000232528
R00367	reaction	S-adenosyl-L-methionine:glycine N-methyltransferase	4.76855 × 10^−5^
R00369	reaction	L-alanine:glyoxylate aminotransferase	0.000230559
R00472	reaction	acetyl-CoA:glyoxylate C-acetyltransferase (thioester-hydrolysing, carboxymethyl-forming)	0.000001
R00489	reaction	L-aspartate 1-carboxy-lyase (beta-alanine-forming)	1.35129 × 10^−5^
R00490	reaction	L-aspartate ammonia-lyase (fumarate-forming)	1.20605 × 10^−5^
R00585	reaction	L-serine:pyruvate aminotransferase	0.000001
R00588	reaction	L-serine:glyoxylate aminotransferase	0.000001
R00610	reaction	sarcosine:oxygen oxidoreductase (demethylating)	0.000001
R00692	reaction	L-phenylalanine:pyruvate aminotransferase	2.4388 × 10^−5^
R00693	reaction	acetyl-CoA:L-phenylalanine N-acetyltransferase	0.000001
R00751	reaction	L-threonine acetaldehyde-lyase (glycine-forming)	0.000001
R00841	reaction	sn-glycerol-3-phosphate phosphohydrolase	2.58173 × 10^−5^
R00842	reaction	sn-glycerol-3-phosphate:NAD+ 2-oxidoreductase	0.000186587
R00846	reaction	sn-glycerol-3-phosphate:oxygen 2-oxidoreductase	1.77887 × 10^−5^
R00847	reaction	ATP:glycerol 3-phosphotransferase	4.73357 × 10^−6^
R00905	reaction	3-ureidopropanoate amidohydrolase	0.000001
R00907	reaction	L-alanine:3-oxopropanoate aminotransferase	0.000001
R00908	reaction	beta-alanine:2-oxoglutarate aminotransferase	0.000105695
R00966	reaction	UMP:diphosphate phospho-alpha-D-ribosyltransferase	5.44821 × 10^−5^
R00974	reaction	cytosine aminohydrolase	0.000186035
R00996	reaction	L-threonine ammonia-lyase (2-oxobutanoate-forming)	0.000001
R01030	reaction	sn-glycero-3-phosphocholine glycerophosphohydrolase	0.000001
R01080	reaction	uridine ribohydrolase	3.75425 × 10^−6^
R01082	reaction	(S)-malate hydro-lyase (fumarate-forming)	0.000001
R01132	reaction	IMP:diphosphate phospho-D-ribosyltransferase	0.000001
R01229	reaction	GMP:diphosphate 5-phospho-alpha-D-ribosyltransferase	0.000001
R01244	reaction	adenine aminohydrolase	0.000001
R01245	reaction	adenosine ribohydrolase	7.60526 × 10^−6^
R01273	reaction	N-ribosylnicotinamide ribohydrolase	0.000001
R01274	reaction	palmitoyl-CoA hydrolase	0.000001
R01280	reaction	palmitate:CoA ligase (AMP-forming)	0.000001
R01310	reaction	phosphatidylcholine phosphatidohydrolase	0.000001
R01312	reaction	phosphatidylcholine cholinephosphohydrolase	1.23257 × 10^−5^
R01315	reaction	phosphatidylcholine 2-acylhydrolase	0.000001
R01316	reaction	phosphatidylcholine 1-acylhydrolase	1.62448 × 10^−5^
R01317	reaction	phosphatidylcholine 2-acylhydrolase	0.000001
R01318	reaction	acyl-CoA:1-acyl-sn-glycero-3-phosphocholine O-acyltransferase	0.000001
R01319	reaction	acyl-CoA:2-acyl-sn-glycero-3-phosphocholine O-acyltransferase	8.27159 × 10^−6^
R01321	reaction	CDP-choline:1,2-diacyl-sn-glycerol cholinephosphotransferase	0.000001
R01388	reaction	D-glycerate:NAD+ 2-oxidoreductase	0.000001
R01392	reaction	D-glycerate:NADP+ 2-oxidoreductase	2.02073 × 10^−6^
R01393	reaction	hydroxypyruvate carboxy-lyase (glycolaldehyde-forming)	0.000001
R01394	reaction	hydroxypyruvate aldose-ketose-isomerase	8.73738 × 10^−6^
R01447	reaction	(S)-lactate:oxaloacetate oxidoreductase	0.000183101
R01561	reaction	adenosine:phosphate alpha-D-ribosyltransferase	0.000001
R01566	reaction	creatine amidinohydrolase	0.000001
R01567	reaction	ATP:thymidine 5′-phosphotransferase	0.000001
R01569	reaction	thymidylate 5′-phosphohydrolase	0.000001
R01570	reaction	thymidine:phosphate deoxy-alpha-D-ribosyltransferase	0.000001
R01664	reaction	2′-deoxycytidine 5′-monophosphate phosphohydrolase	0.000001
R01666	reaction	ATP:deoxycitidine 5′-phosphotransferase	0.000001
R01676	reaction	guanine aminohydrolase	0.000001
R01677	reaction	guanosine ribohydrolase	0.000001
R01768	reaction	hypoxanthine:NAD+ oxidoreductase	0.000001
R01769	reaction	hypoxanthine:oxygen oxidoreductase	0.000001
R01770	reaction	inosine ribohydrolase	0.000001
R01863	reaction	inosine:phosphate alpha-D-ribosyltransferase	0.000001
R01876	reaction	uridine:phosphate alpha-D-ribosyltransferase	0.000001
R01881	reaction	ATP:creatine N-phosphotransferase	2.17302 × 10^−6^
R01883	reaction	S-adenosyl-L-methionine:guanidinoacetate N-methyltransferase	6.24766 × 10^−6^
R01884	reaction	creatinine amidohydrolase	0.000001
R01890	reaction	CTP:choline-phosphate cytidylyltransferase	8.82724 × 10^−6^
R01891	reaction	CDP-choline:N-acylsphingosine cholinephosphotransferase	0.000001
R01969	reaction	deoxyguanosine:orthophosphate ribosyltransferase	0.000001
R02088	reaction	2′-deoxyadenosine 5′-monophosphate phosphohydrolase	0.000001
R02089	reaction	ATP:deoxyadenosine 5′-phosphotransferase	0.000001
R02099	reaction	ATP:deoxyuridine 5′-phosphotransferase	0.000001
R02102	reaction	2′-deoxyuridine 5′-monophosphate phosphohydrolase	0.000001
R02103	reaction	xanthine:NAD+ oxidoreductase	0.000001
R02107	reaction	xanthine:oxygen oxidoreductase	0.000001
R02143	reaction	xanthosine ribohydrolase	0.000001
R02147	reaction	guanosine:phosphate alpha-D-ribosyltransferase	0.000001
R02269	reaction	5,6-dihydrouracil amidohydrolase	0.000001
R02294	reaction	N-ribosylnicotinamide:orthophosphate ribosyltransferase	0.000001
R02297	reaction	xanthosine:orthophosphate ribosyltransferase	0.000001
R02323	reaction	nicotinamide ribonucleotide phosphohydrolase	0.000001
R02324	reaction	ATP:N-ribosylnicotinamide 5′-phosphotransferase	0.000001
R02473	reaction	(R)-pantoate:beta-alanine ligase (AMP-forming)	0.000001
R02474	reaction	pantothenate amidohydrolase	0.000001
R02484	reaction	deoxyuridine:orthophosphate 2-deoxy-D-ribosyltransferase	0.000001
R02485	reaction	deoxycytidine aminohydrolase	0.000001
R02541	reaction	sphingomyelin cholinephosphohydrolase	0.000001
R02542	reaction	sphingomyelin ceramide-phosphohydrolase	1.65697 × 10^−5^
R02556	reaction	deoxyadenosine aminohydrolase	0.000001
R02557	reaction	deoxyadenosine:orthophosphate ribosyltransferase	0.000001
R02719	reaction	xanthosine 5′-phosphate phosphohydrolase	0.000001
R02745	reaction	1-(1-alkenyl)-sn-glycero-3-phosphocholine aldehydohydrolase	2.92384 × 10^−5^
R02746	reaction	1-acyl-sn-glycero-3-phosphocholine acylhydrolase	0.000001
R02747	reaction	2-acyl-sn-glycero-3-phosphocholine acylhydrolase	0.000001
R02748	reaction	deoxyinosine:orthophosphate ribosyltransferase	0.000001
R02814	reaction	oleoyl-[acyl-carrier-protein] hydrolase	1.75613 × 10^−6^
R02922	reaction	creatinine iminohydrolase	1.07937 × 10^−6^
R03018	reaction	ATP:pantothenate 4′-phosphotransferase	1.45508 × 10^−5^
R03315	reaction	ATP:pseudouridine 5′-phosphotransferase	0.000001
R04014	reaction	dodecanoyl-[acyl-carrier-protein] hydrolase	0.000001
R05794	reaction	CDP-diacylglycerol:choline O-phosphatidyltransferase	4.05435 × 10^−6^
R07063	reaction	linoleoyl-CoA,hydrogen-donor:oxygen oxidoreductase	0.000001
R07064	reaction	phosphatidylcholine 2-acylhydrolase	0.000001
R07377	reaction	Phosphatidylcholine + L-Serine <=> Phosphatidylserine + Choline	0.000106528
R07420	reaction	Phosphocreatine <=> Creatinine + Orthophosphate	0.000001
R07859	reaction	Phosphatidylcholine + H_2_O <=> 1-Acyl-sn-glycero-3-phosphocholine + (9Z,12Z,15Z)-Octadecatrienoic acid	0.000001
R07860	reaction	Phosphatidylcholine + H_2_O <=> 2-Acyl-sn-glycero-3-phosphocholine + (9Z,12Z,15Z)-Octadecatrienoic acid	0.000001
R07861	reaction	(9Z,12Z,15Z)-Octadecatrienoic acid + Reduced acceptor + Oxygen <=> Stearidonic acid + Acceptor + 2 H_2_O	0.000001
R08157	reaction	octanoyl-[acyl-carrier protein] hydrolase	0.000001
R08163	reaction	octadecanoyl-[acyl-carrier protein] hydrolase	0.000001
R08174	reaction	stearoyl-CoA hydrolase	0.000001
R08176	reaction	oleoyl-CoA hydrolase	0.000001
R08178	reaction	(9Z,12Z,15Z)-Octadecatrienoyl-CoA + H_2_O <=> CoA + (9Z,12Z,15Z)-Octadecatrienoic acid	0.000001
R08179	reaction	(5Z,8Z,11Z,14Z,17Z)-icosapentaenoyl-CoA hydrolase	0.000001
R08180	reaction	(4Z,7Z,10Z,13Z,16Z,19Z)-Docosahexaenoyl-CoA + H_2_O <=> CoA + (4Z,7Z,10Z,13Z,16Z,19Z)-Docosahexaenoic acid	0.000001
R08181	reaction	gamma-Linolenoyl-CoA + H_2_O <=> CoA + (6Z,9Z,12Z)-Octadecatrienoic acid	0.000001
R08969	reaction	ceramide:phosphatidylcholine cholinephosphotransferase	0.000001
R10836	reaction	AMP:phosphate (5-phospho-alpha-D-ribosyl)transferase	4.57252 × 10^−6^
R10838	reaction	UMP:phosphate (5-phospho-alpha-D-ribosyl)transferase	0.000109354
R11074	reaction	(S)-malate carboxy-lyase	9.72494 × 10^−5^
R11664	reaction	Dodecanoic acid + Malonyl-CoA + Glycine <=> 4-Keto-2-undecylpyrroline + 2 H_2_O + 2 CO_2_ + CoA	0.000001
R12429	reaction	[lipoyl-carrier protein E2]-L-lysine:octanoate ligase (AMP-forming)	0.000001
R12955	reaction	uridine 5′-monophosphate phosphoribohydrolase	2.27237 × 10^−5^
R12958	reaction	dIMP phosphohydrolase	0.000001
R12967	reaction	sarcosine,5,6,7,8-tetrahydrofolate:O_2_ oxidoreductase	2.74303 × 10^−6^
R13029	reaction	sarcosine:ferredoxin oxidoreductase (demethylating)	0.000001
C00037	compound	Glycine	1.58549 × 10^−6^
C00041	compound	L-Alanine	1.54652 × 10^−5^
C00079	compound	L-Phenylalanine	0.000001
C00093	compound	sn-Glycerol 3-phosphate	0.000001
C00099	compound	beta-Alanine	0.000001
C00106	compound	Uracil	0.000001
C00122	compound	Fumarate	0.000001
C00147	compound	Adenine	0.000001
C00149	compound	(S)-Malate	0.000001
C00157	compound	Phosphatidylcholine	0.000001
C00168	compound	Hydroxypyruvate	0.000001
C00188	compound	L-Threonine	0.000001
C00213	compound	Sarcosine	0.000001
C00214	compound	Thymidine	0.000001
C00219	compound	Arachidonate	2.52006 × 10^−6^
C00242	compound	Guanine	0.000001
C00249	compound	Hexadecanoic acid	0.000001
C00262	compound	Hypoxanthine	0.000001
C00300	compound	Creatine	0.000001
C00307	compound	CDP-choline	0.000001
C00366	compound	Urate	0.000001
C00385	compound	Xanthine	0.000001
C00526	compound	Deoxyuridine	0.000001
C00550	compound	Sphingomyelin	0.000001
C00559	compound	Deoxyadenosine	0.000001
C00670	compound	sn-Glycero-3-phosphocholine	0.000001
C00712	compound	(9Z)-Octadecenoic acid	0.000001
C00791	compound	Creatinine	0.000001
C00864	compound	Pantothenate	0.000001
C00881	compound	Deoxycytidine	0.000001
C01530	compound	Octadecanoic acid	0.000001
C01762	compound	Xanthosine	0.000001
C02067	compound	Pseudouridine	0.000001
C02642	compound	3-Ureidopropionate	0.000001
C02679	compound	Dodecanoic acid	0.000001
C03150	compound	Nicotinamide-beta-riboside	0.000001
C03519	compound	N-Acetyl-L-phenylalanine	0.000001
C04230	compound	1-Acyl-sn-glycero-3-phosphocholine	0.000001
C05512	compound	Deoxyinosine	0.000001
C06423	compound	Octanoic acid	0.000001
C06426	compound	(6Z,9Z,12Z)-Octadecatrienoic acid	0.000001
C06427	compound	(9Z,12Z,15Z)-Octadecatrienoic acid	0.000001
C06428	compound	(5Z,8Z,11Z,14Z,17Z)-Icosapentaenoic acid	0.000001
C06429	compound	(4Z,7Z,10Z,13Z,16Z,19Z)-Docosahexaenoic acid	0.000001
C16300	compound	Stearidonic acid	0.000001
